# Testing a Web-Based Parent-Focused HIV Prevention Intervention for Gay and Bisexual Adolescents: Protocol for a Randomized Controlled Trial

**DOI:** 10.2196/81316

**Published:** 2026-06-22

**Authors:** David M Huebner, Brian RW Baucom, Stephanie Micucci, Andrew P Barnett, Maggie Matson, Vincent Guilamo-Ramos, Jenny Mackenzie, Rana Saber

**Affiliations:** 1 Department of Prevention and Community Health Milken Institute School of Public Health George Washington University Washington, DC United States; 2 Department of Psychology University of Utah Salt Lake City, UT United States; 3 Department of Prevention and Community Health George Washington University Washington, DC United States; 4 Psychiatry and Behavioral Health Children's National Washington, DC United States; 5 Feinberg School of Medicine Northwestern University Chicago, IL United States; 6 School of Nursing Johns Hopkins University Baltimore, MD United States; 7 Department of Digital Media Smith College of Engineering & Technology Utah Valley University Orem, UT United States

**Keywords:** HIV prevention, adolescents, sexual minority, gay and bisexual male, parenting, intervention, sexual health

## Abstract

**Background:**

Among US teenagers, 79% of HIV infections are attributable to male-to-male sexual contact; yet, few interventions have been shown to effectively reduce sexual risk among gay and bisexual adolescents (GBA). Parent communication about sex is associated with adolescent sexual risk, and interventions to improve parent communication have been shown to successfully reduce sexual risk among heterosexual samples. However, no interventions designed specifically for parents of GBA have been tested in clinical trials. Parents and Adolescent Talking About Healthy Sexuality (PATHS) is a web-based intervention we created for parents of GBA that aims to improve parent communication about sexuality and HIV and increase parent behaviors supportive of GBA sexual health.

**Objective:**

This trial aims to test whether delivering PATHS to parents of GBA ages 14-19 years will improve GBA sexual health outcomes in the 6 months following intervention delivery. Secondary aims are to test whether the intervention’s effects are sustained at 12 months after the intervention and to examine whether effects are mediated through specific parent behaviors.

**Methods:**

In total, 350 parents of GBA will be recruited online via social media advertising and randomized to receive either PATHS or an active control. PATHS is fully automated, self-paced, and can be completed in a single session lasting under an hour. The active control is an education entertainment film created to provide general support and guidance to parents of GBA. Both parents and their GBA sons will complete online assessments every 3 months over a 1-year period. Primary outcomes will be evaluated at 6 months after the intervention, and then, the control arm will crossover and receive PATHS, and dyads will be followed for another 6 months. Primary outcomes include both adolescent sexual preparedness (eg, condom skills) as well as HIV-related sexual risk behavior (ie, condomless anal or vaginal sex that is not protected by pre-exposure prophylaxis).

**Results:**

The study was funded in March 2022, and we completed enrollment of 393 parent-GBA dyads in September 2025. We project that all participants will have completed study activities by November 2026, with data analysis and results of the trial forthcoming in the first quarter of 2027.

**Conclusions:**

If proven efficacious, PATHS will be among the first HIV prevention interventions shown to reduce sexual risk for GBA. Moreover, as other adolescent-focused interventions emerge, PATHS’ unique focus on parents will offer a complementary, additional means for reaching GBA who do not engage with other intervention options.

**Trial Registration:**

ClinicalTrials.gov NCT05852600; https://clinicaltrials.gov/study/NCT05852600

**International Registered Report Identifier (IRRID):**

PRR1-10.2196/81316

## Introduction

### Background

Youths aged 13-24 years accounted for 19% of new HIV infections in the United States in 2022 [1]. Young men who have sex with men (MSM) make up most of these infections: 82% of new infections among teens ages 13-19 years and 81% of infections among young adults ages 20-24 years [1]. Among young MSM, HIV is further concentrated in African American and Latino youths, who make up 49% and 32% of new infections, respectively [2]. HIV-infected youths are less likely than older individuals to know their HIV status and, as a result, are the group least likely to engage in the continuum of HIV care leading to viral suppression [3]. Less than a quarter of MSM aged 13-19 years have ever taken an HIV test [4,5].

Despite stark disparities in HIV incidence and testing, interventions to reduce sexual risk and increase HIV testing among young MSM remain extremely limited, particularly for MSM in their teens. While a handful of interventions for adolescent men who have sex with men (AMSM) are in various phases of development and testing, all of the emerging interventions we have identified use individual-level approaches to intervene directly with AMSM (eg, SMS text messages and web-based interventions) [6-9], leaving other levels of influence (ie, family, network, and community) largely unaddressed.

An abundance of research with predominantly heterosexual samples demonstrates that parent-adolescent communication about sex is related to various sexual health outcomes, including delayed initiation of sexual activity [10], as well as greater use of condoms [11-14], and other contraceptives [11,13-15]. Emerging research from our team suggests that this is true for AMSM as well [16,17]. Moreover, multiple interventions to promote parent-adolescent communication about sex have been developed and tested in samples comprised of parents of presumably heterosexual adolescents. A systematic review of these interventions found that they were effective at improving multiple dimensions of parent-adolescent communication [18]. In addition to improving communication, a 2019 meta-analysis found that the interventions also had overall positive effects on adolescent sexual health outcomes [19]. Although the positive effects of parent-focused interventions are now well-documented, we note 2 important limitations in this literature. First, none of the trials addressed sexual health outcomes in nonheterosexual youths. Second, we were unable to identify any published report that tested which specific parent behaviors mediated the effects of interventions on adolescent sexual health outcomes.

In response to the dearth of HIV prevention interventions for AMSM, including those with a parent-focus, we created Parents and Adolescents Talking About Healthy Sexuality (PATHS). PATHS is an online intervention delivered to parents of gay and bisexual adolescents (GBA) that aims to increase parent communication about sexuality and HIV as well as other parent behaviors supportive of sexual risk reduction. In a pilot randomized controlled trial (RCT), PATHS was shown to increase both parent and child reports of how much parents discussed HIV, provided instruction on condom skills, helped sons access condoms, and facilitated their sons in getting HIV tests [20].

### Objectives

Building on the promising effects PATHS had on parent behaviors, the goal of this trial is to test whether PATHS improves sexual health outcomes in GBA ages 14-19 years and to examine whether these effects are mediated through specific parent behaviors.

## Methods

### Ethical Considerations

All methods were evaluated for adequate protection of human participants as a part of the National Institutes of Health review process. They were also reviewed and formally approved by the George Washington University Institutional Review Board (NCR213761). All adult participants (parents and GBA ages 18 years and older) reviewed an online informed consent document and indicated their consent electronically. For GBA younger than 18 years of age, parents reviewed an online permission form and provided permission electronically, and their sons reviewed an online assent document and indicated their assent electronically. Participant privacy and confidentiality were maintained in a variety of ways (eg, by using secure online data collection tools; by separating data from identifiers and storing these in secure, password-protected files). Parents received a US $100 gift card after completing the first 3 study requirements: survey 1, engaging in the intervention, and completing survey 2. Parents then received a US $50 gift card after completing each additional assessment. Sons received a US $25 gift card after completing any study activity.

### Study Design

We are conducting an RCT of PATHS with a national sample of parent-GBA dyads recruited online (proposed n=350, 50% racial or ethnic minority). Parents will be randomized in a 1:1 ratio to receive either PATHS or an active control, which is a film-based intervention that provides general support to parents of GBA. The study condition is unblinded for both participants and the research team. Parent and child assessments will occur every 3 months over a 1-year period. Primary outcomes will be evaluated at 6 months after the intervention, and then, the control arm will crossover and receive PATHS, and dyads will be followed for another 6 months. This allows us to further test the effects of PATHS in the control arm while simultaneously modeling the longer-term effects at 9 and 12months in the original intervention arm.

### Participants

Intervention participants will be parents or legal guardians of GBA. Assessment participants will be the GBA sons of the parents enrolled. For inclusion criteria, we operationalize GBA as individuals ages 14-19 years, who are assigned male sex at birth, who identify their current gender as male, and who identify as some nonheterosexual orientation. Additional inclusion criteria include: parents and GBA must cohabitate at least 2 days per week (eg, parents with sons at college, or with irregular custody, will not be eligible), the participating parent must be aware of their son’s sexual minority identity, and GBA must report being HIV-negative or untested. Participation is open only to parent-son dyads in which both members agree to take part, and both must have the capacity to use an internet-enabled device. We will purposively recruit a sample that is at least 50% racial or ethnic minority GBA.

PATHS was created for parents of cisgender male youths, insofar, as it focuses on sexual behaviors that cisgender GBA might engage in and uses terminology that cisgender male youths typically use to describe their physical characteristics and sexual behaviors. Thus, our intention in excluding noncisgender youths was to ensure that the content was relevant to participating parents and to prevent the intervention from suggesting language to parents that would be inappropriate for use with adolescents of other gender identities. However, in the short time since we developed PATHS, adolescents’ understanding and use of gender terminology has evolved rapidly, with increasing number of youths describing their genders using more expansive terms (eg, genderqueer), while simultaneously experiencing little discordance between that identity and their physical sex characteristics (eg, an adolescent assigned male at birth, who identifies as genderqueer, but has no interest or desire in changing anything about their physical body to align with another sex). This became apparent as we initiated recruitment for the study and discovered that many parents identified their son’s current gender as “male” during screening, while the sons identified their gender using a more expansive term. Given this reality, we decided that PATHS would be appropriate for families in which a parent understands their son to be male, but their son identifies his gender more expansively, in the absence of any other indicator that he is on a developmental path toward making any kind of physical transition through gender-affirming care. Thus, we expanded our inclusion criteria to include youths who either (1) are assigned male at birth and identify as male or (2) are assigned male at birth, are identified by their parents as male, self-identify as some other gender, and report no interest in taking any action that would alter their body’s physical characteristics (ie, hormones and surgery).

Parents of GBA are recruited through a variety of means, building on strategies we have used previously to successfully recruit this population [20,21]. We maintain a website that serves as a platform for providing resources to parents of lesbian, gay, bisexual, and transgender youths who we are testing. We drive traffic to the site through multiple means. To advertise new studies, we use paid advertisements on social media platforms (Facebook and Instagram) aimed at specific demographic groups (eg, parents of teens). Additionally, we benefit from our historic work in this area over the past 10 years, as our website is now linked as a resource on other sites (eg, local PFLAG chapters and college counseling centers) that drive traffic to us each month.

Parents who arrive at our website during the study are directed to a brief screening questionnaire that helps identify the most relevant resources for their family and also assesses their eligibility for this study. The automated screener directs only eligible parents to a prerecorded video featuring the study principal investigator (DMH) explaining the purpose of the study and procedures. Interested parents are then directed to the online REDCap (Research Electronic Data Capture; Vanderbilt University) system, a secure, web-based application for survey data collection [22]. Parents provide consent to participate, permission for their son (if he is younger than 18 years of age), and contact information for themselves and their son. Then, they complete their baseline assessment (preintervention) within the REDCap system. Upon completion of the parent assessment, sons are automatically contacted via email or text and invited to participate in an online questionnaire study about “how parents and sons discuss important topics.” Sons are not directly engaged in our intervention and are not informed of the nature of the intervention their parents will receive; these steps decrease GBA resistance to participation (eg, if they want to avoid talking to their parents about sex) and also reduce social desirability bias in their responses (ie, if they understood the intervention goals). Interested sons click a link directing them to the REDCap system, where they provide consent (or assent if younger than 18 years of age) and then complete the baseline assessment. Once parents and sons have completed their initial assessments, the study team receives an electronic alert notifying us that a family is prepared for enrollment in the RCT. At this point, we take a number of steps to ensure that parent-son dyads are legitimate and truly eligible: (1) we first review screener data to identify parents who have completed a screener more than once (eligibility criteria are not advertised, and parents complete the screener naïve to our criteria); parents who have completed the screener multiple times are ineligible unless data from the first assessment indicate that they have multiple children they might have screened for; (2) we next review responses from parent and son assessments to check for consistency (eg, age) and to ensure again that eligibility criteria are met (eg, to ensure that the son has not reported being HIV-positive); and (3) finally, we ask dyads to verify their identities in 1 of 2 ways: either uploading copies of their photo identification or participating together in a brief 5-minute video chat. Families for whom we have concerns about their authenticity or eligibility are required to participate in a video chat to provide clarification about the information they provided. Once verified, parents are considered formally enrolled in the RCT and randomized.

### Randomization

We randomize parents in a 1:1 ratio to receive either PATHS or control at the start of the study; following the 6-month assessment, parents initially randomized to the control group are given PATHS. Initial random assignment is stratified by sons’ report of sexual risk (any vs no prior condomless anal or vaginal sex [CAVS]) and race or ethnicity (non-Hispanic White vs other racial or ethnic backgrounds) and blocked in groups of 20.

### Treatment Conditions

Participants assigned to the intervention condition receive both Lead With Love (LWL) and PATHS after randomization. Participants initially assigned to the control condition receive only LWL.

#### Lead With Love

LWL is a 35-minute “education entertainment” film-based intervention we developed to provide parents with a lesbian, gay, or bisexual child with general support, information about sexual orientation, and behavioral guidance for supporting their child (unrelated to HIV) [21]. Pre- and postdata from parents who view LWL suggest that parents feel greater self-efficacy for being a good parent to a lesbian, gay, or bisexual child after viewing the film [21].

#### Parents and Adolescents Talking About Healthy Sexuality

We developed PATHS through an iterative process involving review of existing theory and empirical research, collecting primary qualitative data from parents and youths, and collaborating with parents to create the intervention content.

**Figure 1:**
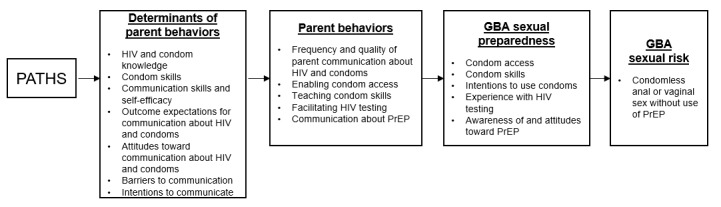
PATHS conceptual model. GBA: gay and bisexual adolescent; PATHS: Parents and Adolescent Talking About Healthy Sexuality; PrEP: pre-exposure prophylaxis.

Existing theory about why parents communicate about sex largely focuses on characterizing barriers to doing so and suggests that interventions must address these barriers to be effective [23,24]. Common barriers include parent discomfort and embarrassment, lack of knowledge of technical aspects of sex, fears of encouraging sex, low self-efficacy to communicate, and concerns about how the child will react and whether communication will matter. No existing theory thoroughly describes parents’ approach-oriented motivations for discussing sex with adolescents, but some suggest applying the integrated behavioral model [18], which we adopted to guide this intervention. When applied to communication, the model broadly suggests that parents discuss sex with their children when parents have intentions to do so, appropriate skills, and limited barriers. Intentions should be driven by self-efficacy to communicate, favorable attitudes toward communication, and positive expectations for how conversations will go. Figure 1 depicts the conceptual model underlying the intervention.

To further understand families’ experiences with communicating about sexuality and HIV, we conducted qualitative interviews and focus groups with families. Using these qualitative data as a foundation, together with content from sexual health communication interventions for parents of heterosexual youths, we developed PATHS in collaboration with a volunteer Board of Family Experts, comprised of 4 parents of AMSM and 6 AMSM. Once a working version of the intervention had been developed, it was tested for usability and clarity with 6 parents of AMSM.

PATHS was developed with the goal of changing the following parent behaviors: (1) to increase the overall frequency and quality of parent-adolescent communication about sex and HIV, including communicating about condoms and pre-exposure prophylaxis (PrEP) as means to prevent HIV; (2) to support parents teaching their sons the proper mechanics of condom use; (3) to help parents enable their sons’ access to condoms; and (4) to assist parents in facilitating HIV testing for their sons. These behaviors, in turn, are hypothesized to increase GBA preparedness for sex and ultimately decrease CAVS that is not protected by PrEP.

Parents access PATHS on any web-enabled device with a unique username and password. PATHS is fully automated, self-paced, and designed so parents can access all content in a single session (lasting 30-60 minutes) or in multiple sessions. Content is divided into seven required modules: (1) welcome and overview, (2) importance of communication, (3) HIV information, (4) using and acquiring condoms, (5) PrEP, (6) HIV testing, and (7) a review with a parent “to-do” list. Optional supplemental modules include content on understanding anal intercourse and what to do if a child tests positive for HIV. These topics were deemed optional because they either did not apply to all youths (ie, what to do if a child tests positive) or because parents might not be ready to receive the material (ie, understanding anal intercourse).

PATHS content focuses on changing the relevant determinants of parent behaviors (Figure 1). Drawing from theory and research pointing to the benefits of multimodal communication [25,26] that includes both peer and expert-delivered messages [27-29], content is communicated via a combination of narrated text pages, animated videos, and videos integrating experiences of real parents, GBA, and adolescent health experts. Video content applies multiple principles of social learning theory [30,31]. To increase parents’ perceived similarity to and identification with the “cast” depicted in the videos, we feature brief personal stories in which our parents and experts describe common experiences others might relate to (eg, a parent sharing their experience of a child coming out or an expert mentioning that they are also a parent). Additionally, the cast is weighted with racial or ethnic minority representation—75% (3/4) of the adolescent health experts are people of color, and over half of the parents or youths depicted are people of color. In addition, consistent with social learning principles, parents in the videos describe their experiences engaging in intervention-recommended behaviors in ways that emphasize both their persistence in the face of barriers as well as the ultimate rewards they gleaned from engaging in the behavior.

Because goal-setting is an effective component of many behavioral interventions [32], each required module was followed by an interactive activity encouraging parents to select how they planned to follow up with their son about the content. Recognizing that parents and sons approach these discussions about sexuality with different degrees of experience and comfort, we suggest 3 different options for how to engage with their son about each segment of the content. The options vary in intensity or complexity and, presumably, in the degree to which the interaction will be impactful for their son. We categorize the options as “good,” “better,” and “best” and inform parents that taking any of the recommended actions will help their son reduce his HIV risk. For example, in working with parents to teach their sons the proper mechanics of condom use, we give parents the following three options: (1) forward an instructional video to your son via the web application with a personalized note (good); (2) forward the video, watch it with your son, and discuss it (better); or (3) do a condom demonstration together with your son (best). The intervention allows parents to facilitate some of these interactions by directly forwarding certain content (eg, videos and fact sheets) to their sons’ mobile numbers or email addresses. Once parents select the option they want to pursue, a personalized to-do list is populated for them that they can print or email to themselves in the final module.

After initially developing PATHS, we conducted a pilot RCT with 61 parent-adolescent dyads. Results found that PATHS increased both parent and child reports of how much parents discussed HIV, provided instruction on condom skills, helped sons access condoms, and facilitated their sons in getting HIV tests [20]. In total, 96% (30/31) of parents in the pilot trial indicated that they were “very” or “extremely” satisfied with PATHS, that it was easy to use, and the content credible. The intervention did not increase family conflict as reported by either parents or adolescents.

#### One-Month Refreshers

One month after completing the initial intervention assigned, parents are invited to revisit the PATHS web application to complete a “refresher” session designed to reinforce the intervention messaging. The refresher used in this trial was newly developed after the initial pilot RCT of PATHS.

For parents assigned LWL alone, the refresher consists of a series of text pages that summarize the important messages and guidance provided in the original film. For parents assigned PATHS, the refresher first asks parents whether they have been able to successfully engage in each of their selected interactions with their son (ie, the activities they chose to put on their “to-do” list). If a parent indicates that they have not achieved their goal in the previous month, they are presented with a list of possible barriers or reasons why completing the activity was difficult for them. After parents select the barriers they experienced, they are provided with content tailored to each selected barrier designed to help the parents navigate the particular challenge. Once parents review that content, they have the opportunity to reset the goals they did not achieve. Parents also receive reinforcement for any goal they did successfully achieve. Goals that were achieved at the “good” or “better” level are reinforced, and parents are given the opportunity to also set a new goal and achieve the “best” status for a given activity (eg, if they previously just forwarded the condom instruction video to their child, they are asked if they would like to try doing a physical condom demonstration with their child). All new goals that parents set for themselves are recorded on a refreshed to-do list that parents can print or email to themselves.

#### Postintervention Nudges

After parents complete different intervention activities, the application automatically sends brief messages to them via text or email to motivate them to engage in behaviors recommended by PATHS (or behaviors recommended by LWL, for participants assigned to the control). Messages are sent 1 week after completion of the initial intervention modules and then again 1 and 2 weeks after completion of the refresher module. For participants who fail to complete an intervention activity, messages are sent at the same intervals, but timed according to when the activity no longer became available to them.

### Assessment Schedule

**Figure 2:**
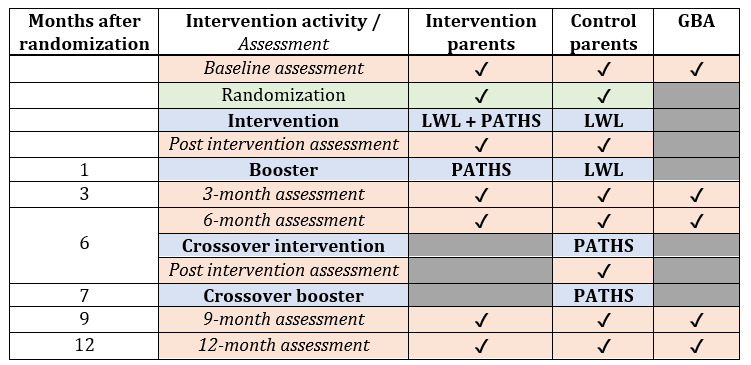
Schedule of activities by participant and condition. GBA: gay and bisexual adolescent; LWL: Lead with Love; PATHS: Parents and Adolescent Talking About Healthy Sexuality. Color coding distinguishes types of study events: intervention activities, assessments, and randomization/crossover procedures.

The schedule of study activities and assessments by participant and condition is summarized in Figure 2.

### Outcome Considerations

Determining the appropriate sexual health outcomes for this intervention trial requires balancing the needs for methodological rigor, feasibility, and developmental appropriateness for the younger adolescents in our study. The primary outcomes for most trials of HIV prevention interventions overwhelmingly fall into two categories: (1) self-reported sexual risk behaviors (eg, condomless intercourse) or (2) biological (eg, laboratory tests for sexually transmitted infections [STIs]). While the rationale for using these outcomes with some populations is strong, they might be less appropriate for adolescents. For example, HIV and STI incidence estimates among younger GBA suggest that powering a trial to detect group differences in these outcomes would require enrolling prohibitively large samples and/or extremely long follow-up periods [33,34]. Moreover, incident STIs and condomless intercourse are of limited utility as outcomes if our hope is to understand intervention effects on youths who have yet to have their sexual debut and who might not have their debut during the study follow-up period.

To address these challenges, we adopt the concept of sexual preparedness (sometimes also referred to as “sexual competence”) [35-38], which is generally defined as the degree to which adolescents have the skills and material resources necessary to engage in sex in a manner that maximizes positive outcomes and minimizes risk. Since PATHS is primarily an HIV-prevention intervention, we focus on those elements of preparedness most relevant to reducing HIV risk: having access to condoms, having the skills to use condoms, intending to use condoms for intercourse, and being “current” on HIV testing, given their level of sexual activity (ie, if not yet sexually active, having had at least 1 experience with testing to understand the process; or if sexually active, having had 1 test in the last year, per Centers for Disease Control and Prevention guidelines). We then use multiple means of assessing these outcomes to increase rigor. Other elements of sexual preparedness (eg, having sexual skills necessary to bring pleasure to oneself or partner and understanding when sex is right for you) might be improved by our intervention (eg, PATHS has content for parents on discussing values with your child as well as information on how to engage in anal sex pleasurably). However, given the limited time we have to engage parents and the importance of sexual risk reduction, those aspects of preparedness are not a primary focus.

Because PrEP is currently clinically indicated only for some adolescents (ie, those who are already sexually active) [39], and because the barriers to PrEP uptake among GBA are numerous and extend well beyond the level of parent influence [40], actual PrEP uptake is not a goal of the intervention at present. Rather, we hope to increase parent and GBA awareness of PrEP, as well as favorable attitudes toward PrEP as an option to consider for sexually active GBA. These outcomes correspond to the first step in the PrEP HIV prevention cascade, which requires that individuals have knowledge of a prevention option and be motivated to use it [41].

Finally, the best measures of behavioral risk for HIV among GBA are still self-reports of sexual behavior obtained with well-tested, developmentally appropriate measures, which we use for this trial [42,43].

### Primary Outcomes

Primary outcomes include GBA’s access to condoms, skills for using condoms, current HIV testing experience appropriate to their level of sexual activity, awareness of PrEP, positive attitudes toward PrEP as a prevention option, intentions to use condoms, and CAVS that is not protected by PrEP. Table 1 summarizes the methods and timing of assessments for each outcome [16,42-51]. CAVS will be assessed using measures developed by Northwestern University’s Institute for Sexual and Gender Minority Health and Well-Being for use with adolescent and young adult MSM [42,43]. The instrument first asks youths about their lifetime sexual experiences broadly (eg, “have you ever done any of the following ...”), and then, directs them to questions that follow up on their experiences (ie, participants with limited sexual experience get questions about kissing and touching and participants with anal or vaginal sexual experience get detailed questions about condom use). This ensures that all participants receive roughly the same number of questions, even if they are less sexually experienced, thereby disincentivizing participants from learning the skip patterns over repeated assessments and misreporting to shorten assessments. Participants who report anal or vaginal sexual experience are queried about the number of partners during the previous 3 months with whom they engaged in anal or vaginal sex with and without a condom. Additionally, participants report detailed information about their 3 most recent anal or vaginal partners (eg, how many times they engaged in receptive anal sex with that partner, with and without a condom). GBA are also queried about their PrEP use, and if they report taking PrEP throughout the previous 3-month period, without missing more than 3 doses a week [52], they are included in the “no CAVS” group.

**Table 1:** Measurement and timing of primary outcomes.

Outcome and reporter			Description of measure		Schedule
**Condom access**					
	GBA^a^	Single item asking if the participant has possession of a condom (at home or elsewhere); if yes, a follow-up question requesting the upload of a photo of the condom in the participant’s hand.		BL^b^, 3, 6, 9, 12	
	Parent	Single item asking the parent if they know their son has possession of condoms.		BL, 3, 6, 9, 12	
**Condom skills**					
	GBA	Condom use self-efficacy scale [44].		BL, 3, 6, 9, 12	
	GBA	Condom knowledge test (objective assessment of ability to differentiate correct and incorrect steps in using condoms from a list) [45].		BL, 3, 6, 9, 12	
	GBA	Video-recorded condom demonstration [47-49].		3, 9	
**HIV testing**					
	GBA	Single item asking if ever received HIV test; follow-up item asking date of most recent test.		BL, 3, 6, 9, 12	
	Parent	Single item asking if son ever received HIV test; follow-up item asking the date of the most recent test.		BL, 3, 6, 9, 12	
**PrEP^c^ attitudes and awareness**					
	GBA	Awareness: single item [16]; attitudes: perception of PrEP (1) efficacy, (2) ease of use, and (3) appropriateness for use in sexually active GBA.		BL, 3, 6, 9, 12	
**Condom use intentions**					
	GBA	Intentions to use condoms for anal or vaginal sex. Three items derived from previous research [50,51].		BL, 3, 6, 9, 12	
**Condomless intercourse**					
	GBA	Past 3-month history of sexual behavior. Count of the number of condomless anal or vaginal sex partners while not adhering to PrEP [42,43].		BL, 3, 6, 9, 12	

^a^GBA: gay and bisexual adolescent.

^b^BL: baseline.

^c^PrEP: pre-exposure prophylaxis.

### Mediators of Intervention Effects: Parent Behaviors

PATHS is hypothesized to have its effect on GBA by increasing parent behaviors that are supportive of sexual health. Their measurement is summarized in Table 2.

**Table 2:** Parent behavioral mediators of intervention effects.

Mediator	Reporter	Description of measure	Schedule
Frequency and quality of parent communication about condoms and HIV	GBA^a^	Perception of parents’ communication behaviors over the prior 3 months. Subscale for frequency or specificity of communication. Subscale for quality of communication (ie, how knowledgeable, trustworthy, and honest a parent is in conversations) [17].	BL^b^, 3, 6, 9, 12
Enabling access to condoms	GBA and parent	Guttman scale constructed by asking whether in the previous 3 months, the parent (1) explained to their son where to get condoms, (2) brought condoms home for their son, and (3) took their son shopping to buy condoms together [53].	BL, 3, 6, 9, 12
Teaching condom skills	GBA and parent	Guttman scale constructed by asking whether in the previous 3 months the parent (1) sent the son any written or video information on how to use a condom, (2) discussed with the son how to put a condom on correctly, and (3) demonstrated how to put a condom on correctly [53].	BL, 3, 6, 9, 12
Facilitating HIV testing	GBA and parent	Guttman scale constructed by asking whether in the previous 3 months the parent (1) helped their son schedule an HIV test, (2) drove their son to get an HIV test, and (3) got tested for HIV together with their son [53].	BL, 3, 6, 9, 12
Having informational PrEP^c^ conversation	GBA and parent	In the past 3 months, has the parent shared information with their son about PrEP (yes or no).	BL, 3, 6, 9, 12

^a^GBA: gay and bisexual adolescent.

^b^BL: baseline.

^c^PrEP: pre-exposure prophylaxis.

### Analytic Plan

#### Hypotheses

The specific questions this study proposes to answer are whether PATHS is efficacious in increasing the degree to which GBA are prepared for intercourse and decreasing their engagement in CAVS 6 months after the intervention. We will further test whether intervention effects persist to 1 year after the intervention, and whether they are mediated by increases in parent behaviors that support GBA sexual health. We hypothesize that GBA whose parents receive PATHS will be more prepared for intercourse and will have fewer CAVS partners, relative to GBA whose parents receive only LWL, at 6 months after the intervention; and when control parents crossover and receive PATHS, we will observe similar improvements in that group. We further hypothesize that families originally assigned PATHS will maintain intervention-related gains 1 year after the intervention. Finally, we hypothesize that intervention effects will be mediated by the specific parent behaviors described earlier.

#### Analysis Overview

Hypotheses will be tested using multilevel models (MLMs), which are appropriate for nested designs, and allow us to use a single combined model to test treatment effects at 6 months as well as duration of treatment effects from 6 to 12 months. MLMs are also a recommended method for modeling mediation in randomized clinical trials [54]. We will estimate MLMs using gold standard methods for conducting intent-to-treat analyses and for handling missing data. Separate models will be run for each outcome variable, with estimation methods and distributional assumptions adjusted accordingly (eg, hierarchical generalized MLMs for dichotomous outcomes). We will use recommended methods for controlling type I error in randomized clinical trials with more than 1 outcome of interest by adjusting for our false discovery rate in all analyses [55].

Moderators of intervention effects are of potential public health interest (eg, is the intervention similarly effective across age and/or ethnic groups?). Powering this trial to formally test for differences across all potential moderators was prohibitive. However, given the value in demonstrating that intervention effects are robust across groups, we will explore whether treatment effects observed in the full sample are similar to those we observe in subgroups differentiated by the following variables: GBA age, GBA sexual debut status at baseline, GBA race or ethnicity, and degree of parent engagement in the intervention content.

## Results

The study was funded in March 2022, and we completed enrollment of 393 parent-GBA dyads in September 2025. We project that all participants will have completed study activities by November 2026, with data analysis and results of the trial forthcoming in the first quarter of 2027.

## Discussion

### Potential Implications of Findings

GBA are the group of teens at highest risk for HIV infection in the United States, but evidence-based interventions to reduce that risk are extremely limited. If we demonstrate that PATHS reduces sexual risk behaviors or improves adolescent preparedness for sex, it will provide a new option for addressing an ongoing public health challenge. Additionally, to our knowledge, PATHS will be the first intervention for GBA that addresses a system of influence beyond the individual level. This is critical because not all adolescents are interested in engaging directly in HIV prevention interventions themselves. Having the option of reaching teens via their parents provides a valuable alternative for the field, ensuring that a greater number of adolescents have support for their sexual health.

This trial also aims to expand the field’s approach to assessing sexual health outcomes for adolescents. Clearly, the goal in any sexual health intervention is to reduce the incidence of poor health outcomes (STIs, HIV, and unplanned pregnancy). In the absence of extremely large samples and long follow-up periods, those outcomes will be difficult to detect among adolescents in any intervention trial. Thus, the widely accepted compromise has been to measure self-reported behaviors that are a clear part of the causal chain to incident disease or pregnancy (eg, condomless sex). However, those behavioral outcomes also carry limitations: self-reports are imperfect, and even when they are assessed reliably, those behaviors also must be common enough to create an opportunity for intervention effects to be observed. Often, the solution to the challenge of low behavioral frequency is for trials to recruit only GBA with histories of risk behavior or significant sexual experience. However, sexual health interventions might have different effects for youths who have already established patterns of sexual behavior, relative to youths who are less experienced. If trials preselect youths already engaging in risk behaviors, we will know less about intervention effects on GBA who have yet to initiate sex or sexual risk. Our approach in this trial is to test effects not only on self-reported behaviors but also on outcomes that can be reliably assessed in any adolescent (eg, photo-verified possession of condoms and demonstrated skill using a condom), regardless of their level of prior sexual experience.

Finally, this trial aims to advance our understanding of how parent-focused interventions achieve their effects. Parent-focused intervention trials rarely publish results from mediation analyses that specify how parent behaviors changed as a result of the intervention and how those parent changes related to improvements in adolescent sexual health. By examining these processes, this trial will help future interventions focus their efforts on the parent behaviors most likely to protect adolescents.

### Limitations

This intervention trial uses “sexual preparedness” as a primary outcome. While this approach has the strengths outlined earlier, we must also recognize its limitations. For example, we cannot know for certain whether youths who are in possession of condoms, and who demonstrate correct condom application skills, will necessarily use them consistently in their future sexual encounters. Longitudinal research does show that youths who use condoms in their first sexual encounter are more likely to use condoms up to 7 years later and are less likely to contract STIs during that time [56]. However, future longitudinal research should identify other early indicators that are reliably associated with subsequent disease or pregnancy in order to expand the list of potential proximal intervention outcomes that reliably predict sexual health outcomes throughout adolescence.

Another limitation of this trial is that not all parents have the time, interest, or ability to participate in a parent-focused intervention. Moreover, not all parents of GBA are open with their parents about their sexual orientation. Thus, this intervention, like any intervention, will not reach every GBA. Our prior research demonstrates that GBA who have disclosed their sexual orientations to their parents are actually more likely to engage in sexual risk behaviors than their peers who have not yet disclosed [17,57], so we are confident that parent-focused intervention can reach youths at risk for HIV and STI infection. Nevertheless, parent-focused approaches will be most effective at reducing population-level HIV risk if they are accompanied by a suite of other intervention options, including those that are offered directly to adolescents themselves, as well as those that seek to create change at broader sociostructural levels (eg, by changing schools, clinical practice, or communities).

Finally, while our cross-over design allows us to test intervention effects in the first 6 months using a traditional “pure” control group that has received no intervention relevant to sexual health, this design comes with compromises in testing the duration of intervention effects at 12 months. Specifically, the estimates of longer-term effects must be made by comparing data from intervention families from 6 to 12 months after the intervention to model-based estimates we make about where control families would be from 6 to 12 months if they had not crossed over and received intervention. Those estimates are based on the data the control families provide from baseline to 6 months. The advantages of this approach are that all families enrolled in the study end up with timely access to an intervention with preliminary evidence of efficacy. It also provides a second opportunity to examine intervention effects in the control group by making a within-subject comparison of data from 6 months prior to intervention to 6 months after the intervention. We characterize the test of duration of intervention effects as a secondary aim, given that the tests of those effects rely on model-based estimates of a control effect, rather than a true control.

### Conclusions

At present, interventions empirically shown to reduce HIV risk for GBA are extremely limited. In this landscape, it will be impossible to truly end the HIV epidemic, as thousands of youths will continue to contract HIV before ever being offered any effective HIV prevention programming. The proposed research aims to help fill this enormous gap. If proven efficacious, PATHS will be one of the first interventions shown to reduce HIV risk and increase HIV testing for GBA. It will also provide a complementary means for reaching those GBA who are unmotivated or unable to engage in HIV prevention interventions directly themselves.

## Abbreviations

AMSM: adolescent men who have sex with men
CAVS: condomless anal or vaginal sex
GBA: gay and bisexual adolescent
LWL: Lead With Love
MLM: multilevel model
MSM: men who have sex with men
PATHS: Parents and Adolescents Talking About Healthy Sexuality
PrEP: pre-exposure prophylaxis
RCT: randomized controlled trial
REDCap: Research Electronic Data Capture
STI: sexually transmitted infection
